# SPC: a SPectral Component approach to address recent population structure in genomic analysis

**DOI:** 10.1101/2025.06.04.25328990

**Published:** 2025-06-05

**Authors:** Ruhollah Shemirani, Gillian M. Belbin, Sinead Cullina, Christa Caggiano, Christopher Gignoux, Noah Zaitlen, Eimear E. Kenny

**Affiliations:** 1 Institute for Genomic Health, Icahn School of Medicine at Mount Sinai, New York, NY, USA; 2 Department of Genetics and Genomic Sciences, Icahn School of Medicine at Mount Sinai, New York, NY, USA; 3 Colorado Center for Personalized Medicine, University of Colorado Anschutz Medical Campus, Aurora, CO, USA; 4 Department of Biostatistics and Informatics, University of Colorado Anschutz Medical Campus, Aurora, CO, USA; 5 Department of Neurology, University of California Los Angeles, Los Angeles, CA, USA; 6 Department of Human Genetics, University of California Los Angeles, Los Angeles, CA, USA; 7 Department of Computational Medicine, University of California Los Angeles, Los Angeles, CA, USA; 8 Department of Medicine, Icahn School of Medicine at Mount Sinai, New York, NY, USA

**Keywords:** GWAS, population structure, principal components, identity-by-descent, spectral graph theory, UK Biobank, heritability, fine-scale population structure, genomic inflation

## Abstract

Population structure is a well-known confounder in statistical genetics, particularly in genome-wide association studies (GWAS), where it can lead to inflated test statistics and spurious associations. Traditional methods, such as principal components (PCs), commonly used to adjust for population structure, are limited in capturing fine-scale, non-linear patterns that arise from recent demographic events – patterns that are crucial for understanding rare variant effects. To address this challenge, we propose a novel method called SPectral Components (SPCs), which leverages identity-by-descent (IBD) graphs to capture and transform local, non-linear fine-scale population structure into continuous representations that can be seamlessly integrated into genetic analysis pipelines. Using both simulated datasets and empirical data from the UK Biobank (N ≈ 420,000), we demonstrate that SPCs outperform PCs in adjusting for fine-scale population structure. In simulations, SPCs explained over 90% of the fine-scale population structure with fewer components, while PCs captured less than 50%. In the UK Biobank, SPCs reduced the inflation of p-values in the GWAS of an environmental-driven phenotype by 12% compared to PCs, while maintaining a similar performance to PCs in height, a highly heritable phenotype. Additionally, SPCs improved rare variant association analyses, reducing genomic inflation (e.g., from 7.6 to 1.2 in one analysis), and provided more accurate heritability estimates. Spatial autocorrelation analysis further confirmed the ability of SPCs to account for environmental effects, reducing Moran’s I for both environmental and heritable phenotypes more effectively than PCs. Overall, our findings demonstrate that SPCs provide a robust, scalable adjustment for recent population structure, offering a powerful alternative or complement to PCs in large-scale biobank studies.

## Introduction

Population structure is a critical factor in statistical genetic studies, as it can confound results if not properly addressed (Lawson et al., 2020; Price et al., 2006). This structure arises from genetic and environmental differences across populations, shaped by historical processes such as migration (Gravel et al., 2013), genetic drift (Nei & Tajima, 1981), selection (Berg et al., 2019; Mathieson et al., 2023; Sohail et al., 2019), and assortative mating (Border et al., 2022). These processes create systematic differences in variant frequencies within and between populations that can violate key statistical assumptions in genetic analysis (Price et al., 2010; Sohail et al., 2019). For example, population structure can violate the assumption of independence among individuals in genetic analyses, such as Genome Wide Association Studies (GWAS), leading to false-positive associations and inflated test statistics (Cook et al., 2020). To mitigate these effects, genetic analysis pipelines often use Principal Components (PCs; Price et al., 2006) and/or (generalized) linear mixed models (GLMMs; Kang et al., 2010). PCs represent major axes of genetic variation derived from dimensionality reduction of a genotype matrix, or the Genetic Relatedness Matrix (GRM), which stores pairwise genetic similarity based on variants. GLMMs complement this approach by simultaneously accounting for population structure and cryptic relatedness by modeling a random effect whose covariance structure is defined by the estimated GRM. Both PC- and GLMM-based strategies have been successfully used to address population structure in analysis involving common variants. However, it is less clear whether these methods will be similarly effective for rare variants and more complex fine-scale population structure (Mathieson & McVean, 2012; Privé et al., 2020; Zaidi & Mathieson, 2020).

Large-scale sequencing studies have revealed patterns of recently arisen rare variants and have shown that they tend to be geographically localized (Fu et al., 2013; Gravel et al., 2011). This fine-scale population structure impacting rare variants has been detected in several populations, including Icelandic (Helgason et al., 2005), British (Cook et al., 2020; Gilbert et al., 2022; Nait Saada et al., 2020), and Japanese (Sakaue et al., 2020), revealing distinct genetic patterns shaped by historical movements over the past few thousand years. However, these studies also highlight a critical challenge: traditional methods to account for population structure struggle to capture this discrete, non-linear population structure occurring within recent timescales. The non-linear relationships and sparsity in the GRM introduced by rare variants can make rare variant PCs sensitive to noise and violate the linear assumptions of PCA, complicating the interpretation of results (Baye et al., 2011; Ma & Shi, 2020; Zaidi & Mathieson, 2020). Likewise, incorporating rare variant information in GLMMs introduces several limitations, including higher computational costs, reduced power compared to common variant counterparts (S. Lee et al., 2014; Wainschtein et al., 2022). These findings underscore the need for novel analytical methods tailored to the unique properties of rare variants in statistical genetic analysis, enabling more accurate modeling of fine-scale population structure and the complex genetic architecture they reveal.

To address these limitations, estimates of pairwise haplotypes shared identical-by-descent, or Identity-By-Descent (IBD), offers a more precise approach for detecting recent fine-scale population structure in large genomic datasets (Shemirani et al., 2021). The length of shared IBD segments provides a direct measure of evolutionary time – longer segments indicate more recent population structure, typically within the past dozen generations (Browning & Browning, 2015). At the population level, the aggregated lengths of IBD segments shared between all pairs of individuals across the genome can be utilized to construct graphs of genetic similarity, where individuals are represented as nodes and shared IBD haplotypes are represented as edges (Belbin et al., 2021). Previous studies have shown the advantages of utilizing IBD data to represent recent population structure through the extraction of clusters of individuals who share IBD haplotypes across the genome (Belbin et al., 2021; Caggiano et al., 2023; Dai et al., 2020). These IBD-based clusters reveal population structure missed by traditional methods, leading to differential associations with health outcomes, unique enrichment of casual rare variants, and distinctive performance of polygenic risk scores compared to clusters identified with common variants alone (Belbin et al., 2021; Johnson et al., 2022).

Here, we introduce SPectral Components (SPCs), a graph theory-driven approach that transform the discrete, localized signals of recent population structure, represented by an IBD graph, into a continuous representation of genetic similarity that can be used in statistical genetic analysis pipelines. Central to our method is a novel application of graph Laplacian transformations (Grone et al., 1990), a fundamental concept in computational fields, such as dimensionality reduction (Kunegis et al., 2010), graph-based machine learning (Si et al., 2014), and network analysis (Shuman et al., 2013), due to their ability to encode graph topology and smooth representations of variations in node features across the graph (Shuman et al., 2013). Previous work has applied spectral approaches to common variant data and demonstrated they can be more robust than PCs in finding hidden population structure compared to GRMs (A. B. Lee, Luca, Klei, et al., 2010; A. B. Lee, Luca, & Roeder, 2010). In this study, we use simulated and empirical data from the UK Biobank project, to explore the efficacy of SPCs in adjusting for the effects of population structure compared to traditional methods. We also explore how these approaches impact the association of rare variants with environmental effects. Our results demonstrate that, by leveraging graph spectral decomposition, SPCs optimally extract patterns of recent population structure that are resilient to the presence of discrete and localized structure in both variant and environmental associations.

## Results

### Overview of Spectral Components (SPC) Calculation

**Figure 1 –:**
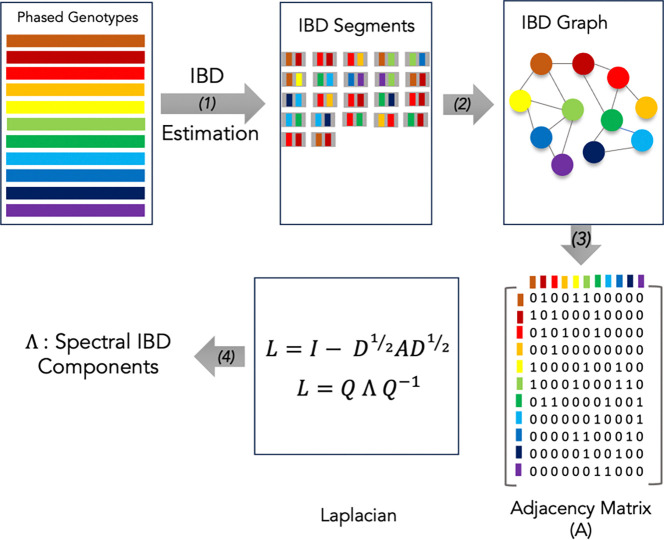
General schema for calculating SPCs. First, phased genotypes are used to estimate segments of genome shared between individuals Identical-by-Descent (IBD). Second, the IBD segments are utilized to generate an IBD relatedness graph with vertices representing participants and edges representing an aggregated IBD sharing between the pairs of participants that passes a threshold of 6cM genome wide. Third, the IBD relatedness graph is transformed into an adjacency matrix (A). Finally, the adjacency matrix A is transformed into its Laplacian form. The eigenvectors corresponding to the smallest eigenvalues of this matrix comprise the set of SPCs.

Previous work has demonstrated the efficacy of identity-by-descent (IBD) graphs in capturing fine-scale population structure signals absent from GRMs (Browning & Browning, 2023; Caggiano et al., 2023, 2023; Zaidi & Mathieson, 2020). We developed a pipeline to extract individuals frequency components underlying a graph of IBD sharing at a population level, or Spectral Components (SPCs; Figure 1). SPCs were calculated in 4 steps. First, we phased genotype data, and estimated IBD using iLASH (Shemirani et al., 2021), We then aggregated the total sums of IBD sharing for each pair of individuals, which were then utilized to generate an undirected, unweighted relatedness graph, with each participant represented as a node. IBD relatedness in this graph was represented as connecting edges between respective nodes, if their sum of IBD sharing surpassed a minimum threshold of 6cM. Thirdly, we generated an adjacency matrix representation of this graph. Lastly, we calculated the spectral components of this matrix as SPCs. To conduct comparative analysis, we further calculated alternative principal components of this graph, along with the principal components of the original genotype data, and rare variants.

### Evaluating Performance to Detect Fine-scale Population Structure on a Simulated Dataset

**Figure 2-:**
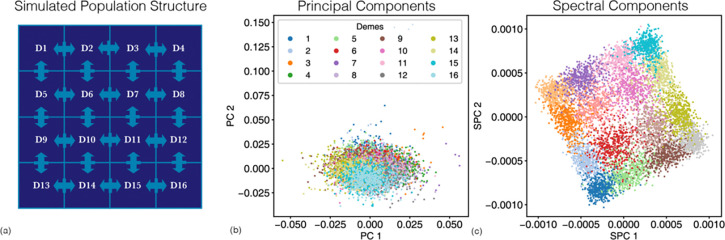
SPCs capture recent population structure in simulations. a) A schematic of our simulation of a cohort with homogeneous population structure with 16 demes and constant migration (5% per generation) between neighboring demes. All demes coalesce to single ancestral deme 200 generations ago. Consequently, simulated individuals have similar genetic background in terms of common variants; simultaneously harboring distinct structure in terms of rare variation and environment of origin. b,c) PC and SPC representation of shared genetic ancestry among simulated samples. Dots in either panel represent samples colored based on the deme of origin. Compared to SPCs, PCs of common variants capture recent population structure inadequately. Their R^2^ scores when predicting vertical and horizontal coordinate of origin for samples (using 25 PCs) were 0.44 and 0.47, respectively. However, the first 8 SPCs, calculated from the same common variant data, had an R^2^ score of 0.90 and 0.91.

To evaluate the performance of SPCs for capturing fine-scale population structure in genomic datasets, we first simulated a population, following Zaidi & Mathieson, 2020. We simulated a 4-by-4 grid of 16 demes with a low migration rate among them that emulates a homogeneous population structure (Figure 2a). This configuration closely resembles the recent fine-scale population structure observed in the White British cohort in the UK Biobank (see Methods). By simulating this nuanced structure, we aimed to evaluate how well SPCs can discern subtle genetic differences that are often overlooked by traditional methods, thereby providing a more detailed understanding of population stratification.

The simulated dataset included 8,000 diploid samples, each with 22 full-length chromosomes. We calculated PCs of common and rare variants. We then down sampled the dataset to retain only the common variants, phased the data to replicate phasing artifacts, and estimated aggregated IBD sharing using iLASH.

Compared to PCs, SPCs demonstrate significant improvements in capturing population structure (Figure 2b–c). We used correlation with deme of origin as a basic measurement of the ability of SPCs to dissect fine-scale population structure. We indexed demes based on their order on horizontal and vertical axes to generate coordinates of origin for each simulated sample, akin to place of birth for an individual. The first 25 PCs explain less than half of vertical and horizontal coordinates of deme of origin for simulated samples (0.44 and 0.47, respectively), while only 8 SPCs were sufficient to explain more than 90% of the variation in the same categories of data. This is reflected in better visual separation between samples based on their deme of origin.

### Evaluating Performance for Disentangling Environmental and Genetic effects

**Figure 3 –:**
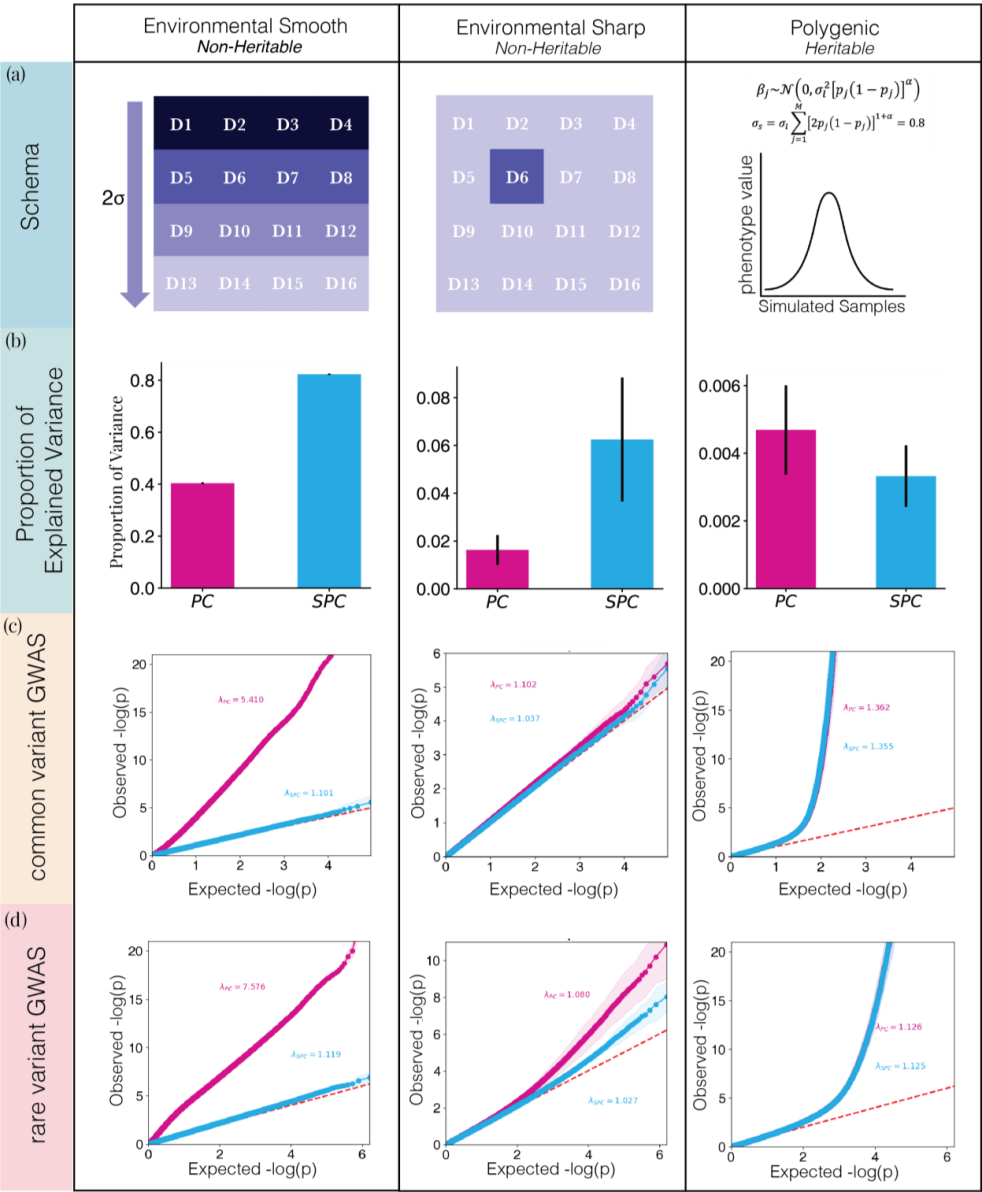
Comparison of PCs and SPCs as covariates to correct for population structure on the outcomes in simulations. **a)** The schema of simulated phenotypes. **b)** proportion of variation in the phenotypes explained by the first 25 PCs and SPCs in 3 categories of simulated phenotypes. SPCs explain a significantly larger proportion of variance for both non-heritable phenotypes; and have a slightly worse performance on the heritable phenotype. The R^2^ score is largest for environmental smooth phenotype due its linear nature compared to the sharp phenotype with PC explaining 39% of the variation and SPC 83%. The error bars represent the standard deviation. Both PCs and SPCs explain less than half a percent of variation in the polygenic phenotype. **c)** Genomic inflation of the results of GWAS analysis of simulated phenotypes using common variants only. The deflation of p-values in environmental smooth phenotype when using SPCs as covariates compared to PCs stems from a higher proportion of variance explained in the phenotype. Consequently, this prevents from any of the p-values reaching genome-wide significance. **d)** Inflation of p-values in a GWAS analysis of simulated phenotypes using uncommon variants (10<MAC<100).

To evaluate the performance of SPCs as covariates in genomic analysis pipelines, we simulated 3 types of continuous phenotypes (Figure 3a), two of which were non-heritable and one that was highly heritable. The first, referred to as ‘environmental smooth’, was a continuous phenotype in which variation solely originated from indirect environmental effects. The mean value of the phenotype decreased from North to South (Figure 3a), and the effects of linear dependencies on deme of origin among the mean values of outcomes were observed. The second, which Zaidi and Mathieson called ‘environmental sharp’(Zaidi & Mathieson, 2020), aimed to investigate the effects resulting from the invalidity of common linear assumptions (Figure 3a). For this phenotype, a single target deme had a phenotype distribution of y∼𝒩(μ,2σ) with a non-zero μ, while other demes had a phenotype distribution of y∼𝒩(0,σ); mimicking non-linear indirect environmental effects. Due to variation stemming only from environmental effects, the expected narrow-sense heritability of both environmental phenotypes simulated is zero.

The third phenotype, on the other hand, is highly heritable (h=0.8), and is affected only by direct genetic effects and random noise. This phenotype, referred to as polygenic, is modeled through the randomized selection of causal variants with effect sizes based on their allele frequency and the total heritability of the trait (0.8). This approach of simulating effect sizes reduces the frequency of high impact variants that undergo significant drift, impacting mean phenotype values per demes drastically, resulting in correlation with deme of birth (Schoech et al., 2019). Absence of such collider effects eliminates both the biases described earlier and the requirement to adjust for them. Thus, fixed-effect covariates, such as PCs, are not effective in addressing possible confounding effects for this phenotype. Instead, Linear Mixed Models are used in such scenarios to account for variance components induced by genetic effects (Price et al., 2010).

To measure the efficacy of SPCs in adjusting for environmental effects on phenotypes, we used proportion of variation in the phenotype explained by covariates (PVE; Figure 3b). In the smooth phenotype, the first 25 SPCs accounted for 82% of the PVE, while the same number of PCs explained only 40%. For the sharp phenotype, the PVE for both models decreased, although the SPCs still explained 3-fold more environmental effects than PCs (6% of PVE vs 2%). Likewise, the score further decreased in the analysis of the heritable phenotype with no environmental effects from recent population structure, with both models explaining less than 0.5% in the variation of the phenotypes, and no statistically significant differences between them. Overall, these simulations demonstrate that SPCs more effectively capture environmental effects compared to PCs, while not impacting estimates of true heritable effects.

### Evaluating Performance for Genome Wide Association Studies

To evaluate how SPCs perform in the context of genome-wide association studies, we conducted GWAS using all three phenotypes, focusing on the distribution of z-scores and the inflation of p-values as a measure of the unadjusted effects of population structure (Devlin & Roeder, 1999; Yang et al., 2011) (Figure 3c). In the GWAS of common variants for the smooth phenotype, the genomic inflation factor (denoted by l) was 1.101 [confidence intervals (CI): 1.084–1.118] when including SPCs in the model, compared to 5.410 [CI: 5.305–5.517] when including PCs in the model. The difference in performance in the sharp model was less pronounced, although statistically significant, 1.102 [CI: 1.066–1.138] for the PC-based models and 1.037 [CI: 1.019–1.054] for the SPC-based ones. This pattern was not observed in the inflation of p–values in the models in the GWAS of the polygenic phenotype, where there were no differences between the inflations of p-values.

To better understand the impact of population structure in the analysis of the polygenic phenotype, we stratified variants into causal and non-causal, referring to variants neither truly associated with the phenotype nor in Linkage Disequilibrium (LD) with causal variants. We assumed that any systematic differences in calculated p-values or effect sizes of non-causal variants in between the two models are likely due to population structure rather than true genetic association. Among the non-causal variants, p-values calculated using PCs were consistently lower (Wilcoxon P-value: 1.54e-53), which suggests they are more affected by confounding. However, the mean effect size estimates for these variants were higher for the SPC models; suggesting the lower p-values in the PC model were not due to higher power, but rather may reflect differences in how population structure is accounted for. For causal variants and variants in linkage disequilibrium with causal variants, there were no differences in estimated effect sizes using SPCs and PCs.

To explore the efficacy of SPCs in adjusting for confounding in rare variant analysis, we conducted GWAS of rare variants with the 3 phenotypes. To increase the power of rare variant GWAS, we simulated a similar dataset with a higher sample count (N=50,000) on a single chromosome (Chromosome 1). GWAS of rare variants showed similar trends to the GWAS of common variants (Figure 3d) across all phenotypes. However, while the genomic inflation of PC model increased significantly to 7.576 [CI: 7.514–7.639] in the analysis of the smooth phenotype, the genomic inflation of the SPC model exhibited a subtle increase to 1.119 [CI: 1.114–1.124]. These results demonstrate the robustness of SPC to site frequency spectra in GWAS analysis.

We next compared SPCs against alternative strategies to calculate fixed-effect covariates to adjust for recent population structure. Previous work by Zaidi and Mathieson demonstrated that PCs derived from rare variants can partially alleviate the limitations of common PCs (Zaidi & Mathieson, 2020). In our simulations, SPCs outperformed PCs of rare variants both in terms of predictive performance (R^2^ score) and decreasing the inflation of p-values. While PCs of rare variants had a higher R^2^ score with the environmental phenotypes compared to PCs of common variants, their performance was consistently lower than that of SPCs (Supplemental Figure 1).

Additionally, principal components based on the IBD relatedness matrix were previously used to improve representation of recent population structure compared to PCs in simulations (Zaidi & Mathieson, 2020). In line with those findings, we explored the alternative strategies for the calculation of continuous covariates using IBD relatedness matrix to compare against SPC. We calculated 11 sets of covariates from IBD relatedness data. SPCs outperformed other covariates both in our simulated analysis and a random subset of UK Biobank (N=50,000) (Supplemental Figures 1 and 2). Our results demonstrate that SPCs provide a more accurate adjustment for recent population structure than alternative covariate strategies (further details in Supplemental Material).

Finally, we evaluated the impact of SPCs on estimating narrow-sense heritability, the proportion of phenotypic variance that is due to a genetic component (Yang et al., 2017), where adjustment of phenotype values using PCs is a common measure to lower the inflation of estimates due to population structure (Abdellaoui et al., 2019; Cook et al., 2020). We calculated the narrow-sense heritability of the smooth phenotype using the GREML method implemented in the GCTA software package (Supplemental Figure 3). This resulted in a heritability estimate of 1.00, indicating the phenotype is fully heritable genetically. Adjustment using PCs of rare variants resulted in a heritability estimate of 0.45. However, adjustment using SPCs resulted in a 12-fold decrease of the estimate to 0.08, suggesting a significantly lower genetic heritability component, which is in line with the non-heritability of the phenotype. This illustrates the optimal effectiveness of SPCs against PCs when analyzing phenotypes with strong environmental components.

To test for overcorrection of genetic factors that leads to the underestimation of narrow-sence heritability, we calculated the heritability of the polygenic phenotype with an estimated heritability of 0.8. The uncorrected estimated heritability for this phenotype, calculated using common variants, was 0.43 (std:0.04). The estimated heritability after correcting for SPCs did not significantly change (0.42-std:0.04). However, correcting for PCs decreased the heritability estimate to 0.36 (std:0.04) (Supplemental Figure 4). Together, this suggests that SPCs have advantages over PCs in accurately estimating heritability in the context of population stratification.

### Analysis of the UK Biobank

We then sought to evaluate the performance of SPCs in real data using genetic data from individuals with self-reported White British ancestry in the UK Biobanks (N~420,000). We focused on two continuous phenotypes: Easting coordinates of birthplace (eastings), which display a linear geographical cline and have negligible heritability, and height, which is established to have high heritability (Figure 3a). In terms of proportion of variance explained, we saw similar results as the simulated data, with SPCs outperforming PCs for both phenotypes. The difference was particularly significant for eastings. The GREML narrow-sense heritability estimates revealed similar patterns, though the gap in estimates was smaller compared to the simulated analysis. The heritability analysis for height resulted in the same estimates in both models (0.7±0.01), while the estimates for eastings were 0.68 (std: 0.01) and 0.04 (std:0.01), using PCs and SPCs, respectively. These results for easting and height are similar to those of environmental smooth and polygenic phenotypes in the simulations, respectively.

Next, we conducted GWAS for both phenotypes to measure the inflation of the test statistics due to population structure, using the first 25 PCs and SPCs, age, age squared, sex, and an age/sex interaction variable, as covariates. To account for indirect genetic effects, and cryptic relatedness, which were not present in the simulated phenotypes, we used the BOLT-LMM software, which applies a Bayesian Shrinkage Prior to adjust for those effects (Loh et al., 2015). In the GWAS of eastings, SPCs resulted in a lower inflation of p-values, as evidenced by a lower genomic inflation factor compared to PCs: 1.200 and 1.369, respectively. In contrast, the GWAS of height showed no significant differences in the inflation of p-values as both studies yielded the same inflation factor (figure 3c). We then conducted a GWAS of rare variants using imputed genotype data, using BOLT-LMM. Looking exclusively at the imputed rare variants, we observed lower genomic inflation factor values for both models in both phenotypes along with similar patterns as the non-imputed GWAS, with SPCs having a lower inflation factor (1.147) compared to PCs (1.311) in easting and both models having the same inflation factor for height.

We then sought to explore the differences between the effect sizes estimated in each model. LD score regression found identical heritability estimates from both models in height and eastings, with a higher intercept for PCs in eastings GWAS. This suggests the difference in performance in eastings was mostly driven by population structure confounding in the PC model (Bulik-Sullivan et al., 2015). In height, where aggregate analysis revealed negligible differences, we extracted the list of loci for which the estimated single variant effect sizes differed significantly. We found that the minor alleles in these loci tended to be more uncommon, compared to all tested variants (Kolmogorov-Smirnov test p-value:5 × 10^−83^), indicating the differences between the two models in height GWAS, thus, is mostly present among uncommon or rare variants. The same pattern was also observed in the results for the eastings GWAS.

We calculated spatial autocorrelation of the phenotypes, in the form of Moran’s I, as a measure of indirect environmental effects (Abdellaoui et al., 2019). A previous study showed the spatial autocorrelation of phenotype distributions is reduced after adjustment using PCs, illustrating the efficacy of PC adjustment in addressing confounding due to such effects (Abdellaoui et al., 2019). Here we replicated that experiment and added SPC adjustment (Figure 3e) as another comparison approach. The Moran’s I for eastings decreased from 0.52 to 0.41 after adjustment using PCs. For height, it decreased from 0.32 to 0.14. Adjustment using SPCs was able to decrease Moran’s I further to 0.38 for easting, and 0.10 for height, indicating additional correction for population structure compared to PCs. In summary, our analysis demonstrates that SPCs consistently outperform PCs in reducing population structure confounding, particularly for phenotypes with strong environmental components, and show comparable performance for highly heritable traits like height, especially when considering rare variant effect sizes and spatial autocorrelation.

## Discussion

We introduced SPectral Components (SPCs), a novel approach that leverages IBD graphs to capture localized, non-linear fine-scale population structure, and transforms them into a continuous representation for statistical genetics analyses. This method overcomes some limitations of Principal Components (PCs), which are optimized for modeling continuous linear ancestral relationships, but struggle to fully account for recent demographic events that shape patterns of rare variation. Our results demonstrate that SPCs outperform PCs, explaining over 90% of fine-scale population structure in simulations while reducing genomic inflation in genome-wide association studies (GWAS) for both environmental and heritable phenotypes. Notably, SPCs improved rare variant association analyses by reducing genomic inflation, while providing more accurate heritability estimates and avoiding overcorrection for the highly heritable trait of height. Spatial autocorrelation analysis further confirmed that SPCs better control confounding from environmental effects, reducing Moran’s I more effectively than PC. These findings establish SPCs as a powerful and scalable tool to improve population structure adjustment and confounding control in large-scale, diverse biobank studies.

This work builds on previous research on IBD- or rare variant-based GRMs which has shown that the latter models can often overcorrect for population structure, leading to loss of true signal in genetic analysis. For example, several studies (Browning & Browning, 2015; Evans et al., 2018) demonstrate that overcorrection can occur because IBD GRMs disproportionately emphasize recent, localized relatedness, which can obscure genuine associations. Additionally, it extends prior applications of spectral approaches to common variant data, which demonstrated they can be more robust than PCs in finding hidden population structure (A. B. Lee, Luca, Klei, et al., 2010; A. B. Lee, Luca, & Roeder, 2010). However, those applications use variants that are shared identical-by-state, rather than haplotypes shared identical-by-descent, which lack genealogical context and are expected to have poorer resolution of confounding for recent population structure and rare variant association. The distinction between fixed effects and random effects on these models further complicates the adjustment process. Fixed effects, like PCs, explicitly control for population structure by removing specific sources of variation, whereas random effects models, like those incorporating GLMMs, aim to partition variance across all genetic relationships, but may inadvertently attenuate rare variant signals by treating them as background noise. SPCs provide a middle ground by capturing fine-scale population structure without overcorrection, using a fixed-effect approach tailored to discrete, localized signals, while maintaining the broader interpretability of linear mixed models. Moreover, the use of SPCs showed a clear reduction in genomic inflation for rare variants, therefore providing a significantly more robust adjustment. Other work on alternative approaches, such as using ancestral recombination graphs (ARGs) to infer genome-wide genealogies that can be combined with GLLMs to improve to power for rare variant association has recently been shown to scale to large biobank datasets (Nait Saada et al., 2020; Zhang et al., 2023). While these approaches show promise, they currently have some limitations, such as implicating large genomic regions in variant association and requiring methodological improvements to LMM scalability. Future work could explore the use of PCs, ARGs, and SPCs in tandem as a powerful tool for addressing the challenges posed by population structure in modern genomics (Ma & Shi, 2020; Zaidi & Mathieson, 2020).

Another focus of this study was to examine how SPCs capture environmental effects, which often overlap with recent population structure (Zaidi & Mathieson, 2020). The increased accuracy of SPCs compared to PCs is especially important given the increasing recognition of environmental confounding in GWAS, where geography and patterns of recent demography can lead to spurious associations. In this context, SPCs explain a significantly greater proportion of variance for environmental phenotypes, such as easting coordinates. This result aligns with earlier work (Abdellaoui et al., 2019; Cook et al., 2020; Leslie et al., 2015; Zaidi & Mathieson, 2020), highlighting the difficulty of decoupling environmental and genetic factors in geographically clustered populations. Our findings support this previous work, demonstrating that population structure confounding remains a major challenge even in large-scale homogeneous cohorts like the White Britons from the UK Biobank. We showed this for the highly polygenic trait of height, as demonstrated by measuring the spatial autocorrelation in the form of Moran’s I. SPCs were able to further decrease the spatial autocorrelation of height compared to PCs. Our results also support the use of SPCs in heritability analysis, where adjustment using SPCs reduced overcorrection of genetic heritability estimates accounting for environmental confounding. By improving adjustment for the environmental confounding effects, SPCs provide a critical advance in the accuracy of genomic analysis for phenotypes that are heavily influenced by recent demography or environmental variables.

We highlight several limitations to this study and directions for future development. First, although we have shown in simulation and empirical data the improvement of SPCs over PCs for large-scale genetic analysis, additional work is needed to further validate the power of SPCs across different datasets and phenotypes. Second, our study was restricted to unrelated samples of White Briton descent in the UK Biobank, and we expect SPC’s to be susceptible to issues in more diverse datasets related to relatedness, admixture, and population differentiation that impact other genetic analysis. Third, further studies could also benefit from extending the application of SPCs to such data and employing methods like burden testing or Generalized Additive Models for more complex association models. Similarly, while we focused on GWAS and heritability estimation, SPCs could also be tested in other genomic analyses, including Polygenic Risk Score calculation, to assess their ability to improve prediction accuracy and calibration. Finally, in recent years the inclusion of non-European populations in genomic resources has increased, yet the challenges of adjusting for more complex population structure deriving from processes of admixture remains significant. The ability of SPCs to detect subtle ancestry differences suggests that this method could improve the accuracy of GWAS in diverse populations, but further work is needed.

In summary, SPCs offer a robust, continuous alternative to PCs for adjusting for recent population structure in genomic studies. By capturing fine-scale, non-linear demographic patterns, SCPs provide more accurate adjustment for confounding due to environmental effects and recent ancestry, particularly in large-scale cohorts. As large-scale biobanks continue to grow in size and diversity, the impact of rare variants in genetic analysis will become increasingly pronounced. In this context, tools like SPCs will be essential for ensuring accurate results, particularly as researchers delve deeper into the genetic architecture of rare variants on common disease and traits influenced by population-structured dynamics. By addressing the combined challenges of population structure, environmental confounding, and rare variant associations, SPC-based approaches pave the way to uncover novel insights into complex disease architecture.

## Methods

### Calculating Spectral and Principal Components

The calculation of SPCs can be described in 4 main steps (Figure 1).

*Step 1:* We conducted QC and phasing on array data. The phased data was used by the IBD estimation algorithm, iLASH, to detect pairwise for IBD for each dataset. An in-depth explanation of genotype QC and phasing, along with details about running iLASH and estimating IBD is described in Shemirani et al., 2021. The results of the first step can be represented as S the set of all IBD segments, long enough to be detected. Each segment has a starting point and an end point $s_{1},$ $s_{2}$, located on autosomal chromosome k.


$$S=\left\{\left(a,b,s_{1},s_{2}\right)|a,b\in N\wedge s_{1},s_{2}\in L_{k}\wedge k=1,\ldots ,22\right\}$$

*Step 2:* the aggregated sums of pairwise IBD sharing (i.e. the total or combined length of IBD haplotypes that are shared per pair of individuals) were calculated and used to generate an IBD relatedness graph. On this graph, nodes represented participants and edge weights represented the aggregated IBD sharing. A minimum threshold of aggregated IBD sharing is enforced both as a QC measure and to reflect the population structure of the dataset.


$$\;G_{IBD}=(V,E)\;\;V=N\;E=\left\{(a,b)|a,b\in N\wedge \sum_{\left(s_{1},s_{2}\right)\in s_{a,b}}\;\left(s_{2}-s_{1}\right)>l\right\}$$

To calculate SPCs we used a threshold of 6cM. However, we explored two other thresholds of 10cM and 15cM for comparison.

*Step 3:* The n×n adjacency matrix A was generated, representing the relatedness graph, where rows and columns both represent n participants in the dataset. Each element $a_{ij}$ in this matrix is equal to 1 if the aggregated length of IBD segments shared between the two participants i and j on the graph is above the threshold of l=6cM; otherwise, the value of the elements is set to zero.


$$a_{ij}=\left\{\begin{array}{l} 1\;\mathrm{if}\;(a,b)\in E\\ 0\;\mathrm{otherwise} \end{array}\right.$$

We refer to this matrix as the binary IBD relatedness matrix since. We also explored the use of non-binary relatedness matrices for comparison for their similarity with common Genetic Relatedness Matrices (GRM).

*Step 4:* Any graph adjacency matrix can be transformed into a Laplacian form (Shuman et al., 2013). Here we defined a symmetrically normalized Laplacian:

$$L^{\mathrm{Sym}}:=I-{\left(D^{+}\right)}^{\frac{1}{2}}A{\left(D^{+}\right)}^{\frac{1}{2}}$$

Where I is the identity matrix, D is defined as the degree matrix, a diagonal matrix where each element $d_{ii}$ is equal to the total number of neighbors for participant i, and $D^{+}$ is the (Moore-Penrose) inverse of D. The Eigenvectors of the Laplacian matrix are commonly referred to as the spectral components of that graph. Briefly, SPCs are the spectral components of the binary IBD relatedness graph with a cut-off threshold of 6cM. Thus, akin to PCs, SPCs attribute a set of numerical values to each vertex based on its projections on the set of principal axes of variation in the graph. The level of detail represented by each axis depends on the corresponding eigenvalue association with it. Eigenvector associated with the smaller eigenvalues will assign similar number to neighbors on the graph. As the associated eigenvalue increases, similar values are assigned to participants that are incrementally further from each other on the graph (further details in Supplemental Material).

We have implemented this pipeline as a Python library that transforms flat text IBD graph file to SciPy sparse matrices and uses CPU or GPU acceleration to calculate SPCs using the same library.

### Generating simulated data

We used the MSprime python software library to implement the simulation pipeline to ensure the distribution of IBD connections and their length is concordant with real datasets (Baumdicker et al., 2022). Following guidelines on simulation of homogeneous population with recent structure akin to that of UK Biobanks (Zaidi & Mathieson, 2020), our pipeline simulated N-by-N grids of demes with constant migration rate of 0.05. We set N=4 (16 demes) for our main simulation with the 22 chromosomes and 8,000 diploid individuals and N=5 for the secondary simulation with one chromosome and 50,000 diploid individuals. The length of the chromosomes in the 8,000-sample simulation were adopted from the hapmap project. Chromosome specific recombination rates were adopted from the stdpopsim library metadata (Adrion et al., 2020). In the 50,000-sample simulation, we used the same information only for chromosome 1, with 248,956,422 basepairs and recombination rate of 1.149 × 10^−8^. The migration rate of 0.05 has been shown to establish demes with average divergence, measured by *F*_*st*_, equal to that of neighboring administrative counties in the UK Biobank dataset (Leslie et al., 2015). All demes originate from a single ancestral 300 generations ago. The first 150 generations going back from the current day were simulated using Discrete Time Wright-Fisher (DTWF) model, implemented in the MSprime library to ensure IBD length distribution is similar to that observed in human genotype datasets. The simulation pipeline is available publicly on Github.

While calculating true IBD data is readily possible with MSprime, we sought to replicate common noise present in real-world applications, such as switch errors and false-positive IBD segments. The simulated tree sequences were converted to whole-genome sequence data in PLINK 1.9 format in order to delete the ground-truth phase information. The data was then filtered to retain only common variants (MAF>1%), and then phased using Eagle v4.2. IBD segments were estimated using the iLASH software. For the comparative analysis of downstream applications, 3 types of phenotypes were simulated (Figure 3a). The first category, referred to as environmental smooth phenotypes, are non-heritable phenotypes with a distribution that depends on the deme of origin. All values were generated using a normal distribution with constant variance across all demes. Moving vertically from top to bottom, the mean of the normal distribution decreases monotonically per row. It decreases from a value of 2σ, for the top row, to zero, for the bottom row. Briefly, in a 5 by 5 grid simulation, this means the phenotype values on the top row are drawn from the distribution y∼𝒩(2σ,σ), the values on the second row are drawn from the distribution y∼𝒩(1.5σ,σ), each subsequent row has a mean that is 0.5σ smaller than the previous, with last row having a mean of zero. Second phenotype, called the environmental sharp phenotype is also non-heritable with a normal distribution with a non-zero mean (y∼𝒩(μ,σ)) at the target deme and a zero-centered normal distribution (y∼𝒩(0,σ)) in all other demes. For the last phenotype, which we refer to as the polygenic phenotype, and which is designed to be heritable, causal SNPs were chosen randomly from each window of 1 KB along the genome with effect sizes drawn from $\beta_{j}∼𝒩\left(0,\sigma_{l}^{2}{\left[p_{j}\left(1-p_{j}\right)\right]}^{\alpha }\right)$ where α is chosen based on the total heritability of phenotype using $\sigma_{s}=\sigma_{l}\sum_{j=1}^{M}\;{\left[2p_{j}\left(1-p_{j}\right)\right]}^{1+\alpha }=0.8$. We then used the weights along with the simulated whole genome sequence data, and the score calculation functionality in the PLINK software to generate the mean phenotype values for each individual.

### UK Biobank Data

Phased genotype data along with the imputed data for the UK Biobank project was downloaded through the accession code 84541, The UK Biobank participants were genotyped using the Applied Biosystems UK BiLEVE Axiom Array by Affymetrix 807,411. The genotype data was phased using the SHAPEIT3 software and the 1000 Genomes Project as the reference panel (Bycroft et al., 2018). After removing indels from autosomal chromosome, a total of 655,532 SNPs were retained. Data field 21000, based on self-reported race/ethnicity was used to extract (N=471,931) individuals with White British ancestry. Information on allele frequency and missingness in both phasing and QC process for GWAS data is available in Loh et al., 2018. We estimated fine-scale IBD sharing using the iLASH software (Shemirani et al., 2021). Second degree relatives were identified using KING. From each pair of these relatives, at least one individual was excluded from the analysis (N=405,508). Height information was accessed using data field 20 (N=352,254). Easting coordinates of birth was accessed using data field 129 (N= 343,845). PCs were calculated using PLINK2 software.

### Genome-wide association study

The GWAS of simulated data was conducted using the GCTA software with default parameters and FastGWA option to run linear regression (Jiang et al., 2019). For the common variant analysis, we excluded variants with MAF<1% using PLINK2 software. For the rare/uncommon variant analysis we excluded variant with MAF>1% using the same software. The GWAS in UK Biobanks was conducted using the BOLT-LMM software version 1 to account for cryptic relatedness and direct genetic effects, absent in the simulated phenotype analysis. Age, Age squared, age, sex, age by sex, and age squared by sex were used as covariates (Loh et al., 2015). The LD reference panel was calculated using genotype data from individuals with reported European ancestry in the 1000 Genomes Project. The default genetic map file calculated for Human Genome version 19 (hg19) was used for genetic distances. Minor allele frequency of 0.001 and a minimum INFO score of 0.3 filters were used for the imputed data.

## Supplementary Material

Supplement 1

## Figures and Tables

**Figure 4 -:**
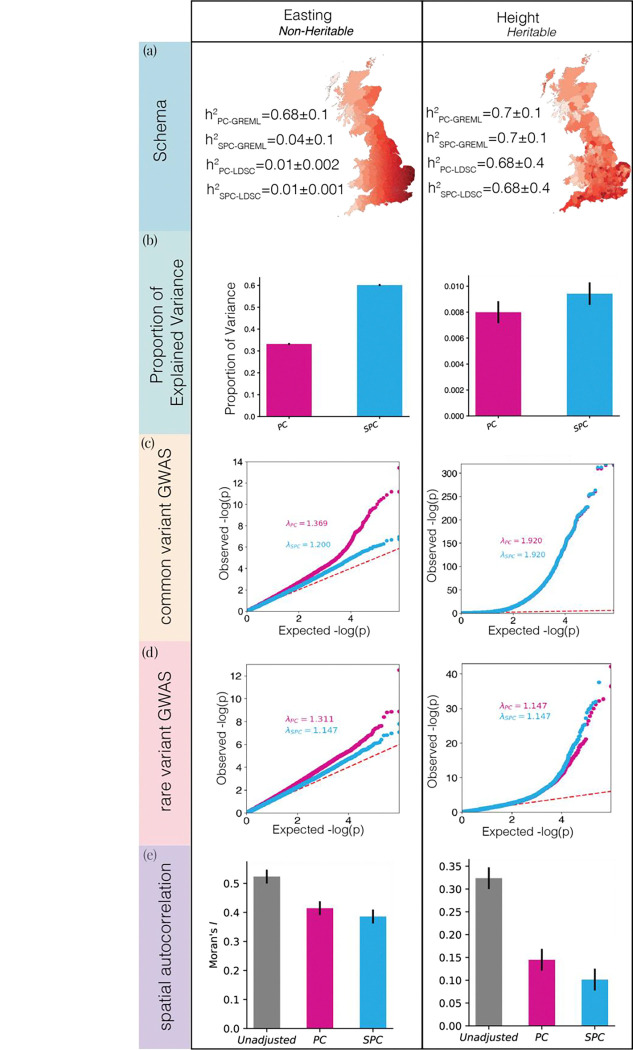
Comparison of PCs and SPCs as covariates to correct for population structure on the outcomes in the UK Biobank. a) The structure of each phenotype. First, easting coordinates of birth is an environmental smooth phenotype with low heritability. GREML heritability estimation after correcting for structure using PCs and SPCs demonstrated the ability of SPCs in modeling recent population structure. Heritability estimation using LD score regression (LDCS) resulted in similar heritability values; with lower LDSC intercept in SPC analysis showing reduced effect of population structure on inflation of Z-scores. Second, Height was chosen as an example polygenic phenotype with high heritability. Heritability estimation resulted in the similar values for both GREML and LDSC. b) Proportion of explained variance in each phenotype by a model that only includes PCs or SPCs as covariates. Results follow a similar pattern to the simulated results. c) Distribution of p-values in the GWAS of common variants for each phenotype that measure the ability of covariates to deal with the effects of recent population structure on the outcomes. d) Distribution of p-values in the GWAS of uncommon variants (MAF < 1%) in the imputed dataset. e) Spatial autocorrelation of each phenotype measured in terms of Moran’s I as a comparison of the ability of each set of covariates to correct against the artifacts of environmental effects.

## Data Availability

UK Biobank data is available for download through application from https://www.ukbiobank.ac.uk LD score reference data was downloaded from https://broad-alkesgroup-ukbb-ld.s3.amazonaws.com Simulated genotype data, along with phenotypes is available on Mendeley repository: 10.17632/9dcpdbzv4m.1
