# Supplementary material for: SPC: a SPectral Component approach to address recent population structure in genomic analysis
Source: medRxiv. 2025 Jun 5:2025.06.04.25328990. Preprint. [Version 1] doi: 10.1101/2025.06.04.25328990 (PMC12155035; doi:10.1101/2025.06.04.25328990)
Supplement: Supplement 1 [file media-1.pdf]

Supplementary Information for “SPC: a SPectral Component approach to address recent population structure in genomic analysis”

Supplemental Figures

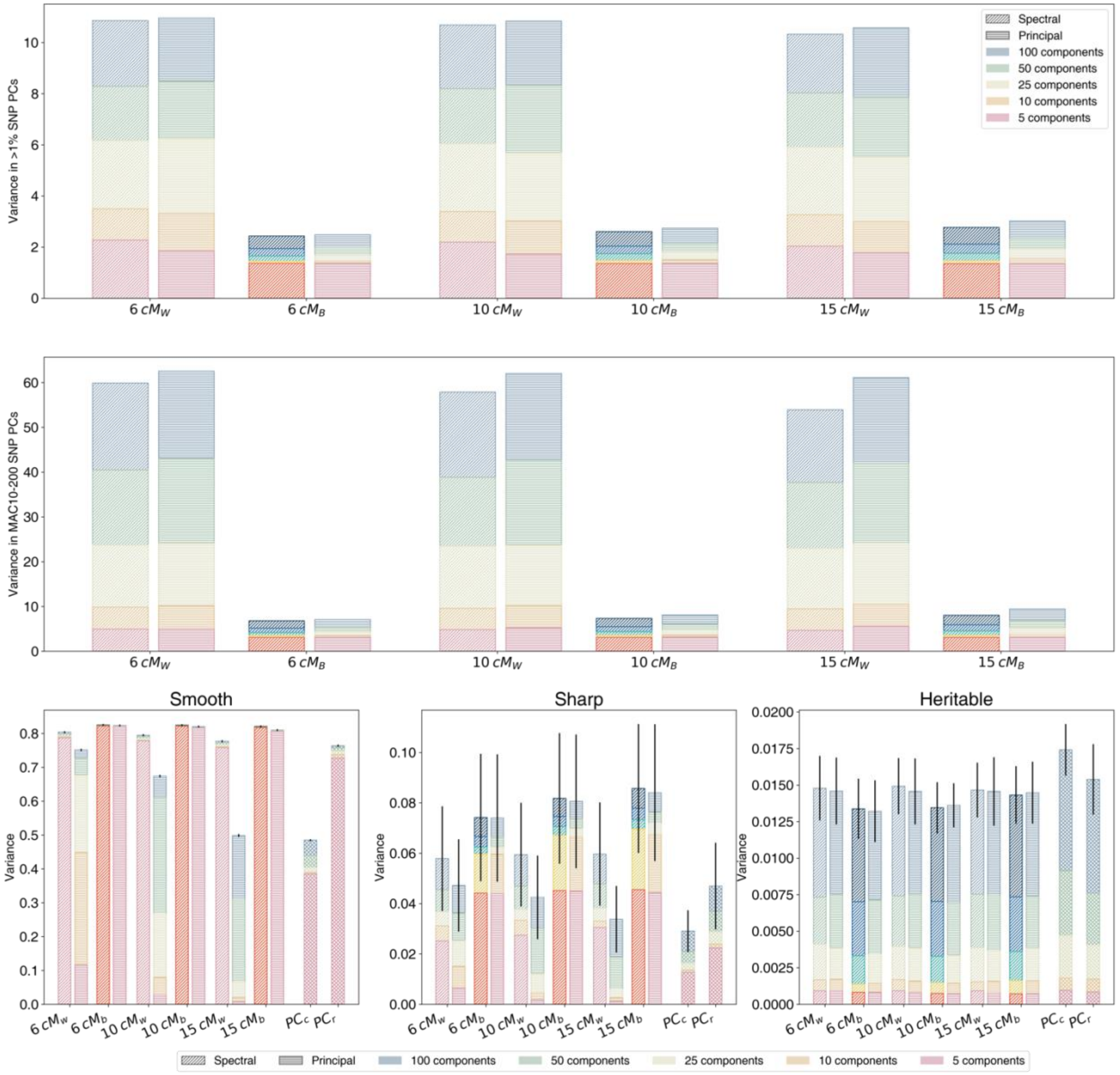

Supplemental Figure 1 – Proportion of variance explained by principal components in simulated phenotypes and PCs of variants. SPCs are highlighted in brighter colors. PCs of common and rare variants are subscripted as PC_C and PC_R, respectively. A and B) Comparison of the proportion of variation in PCs of common (MAF > 1%) and uncommon (10<MAC<200) variants explained by principal components of the IBD graph in the form of the weighted sum of R^2 scores. Principal components of the IBD graph perform better at predicting the variation in uncommon PCs.

Weighted graphs explain a larger proportion of variation in the PC space. SPCs are colored in brighter colors. C) proportion of variation in the phenotypes explained by the principal components in 3 different categories of phenotypes. Spectral components, including SPCs, perform equally or better than principal components of the IBD graph; especially if the graph is weighted. Compared to PCs, IBD-derived covariates explain a larger proportion of variance for both non heritable phenotypes; and have a similar performance on the heritable phenotype, especially for the first 25 principal components.

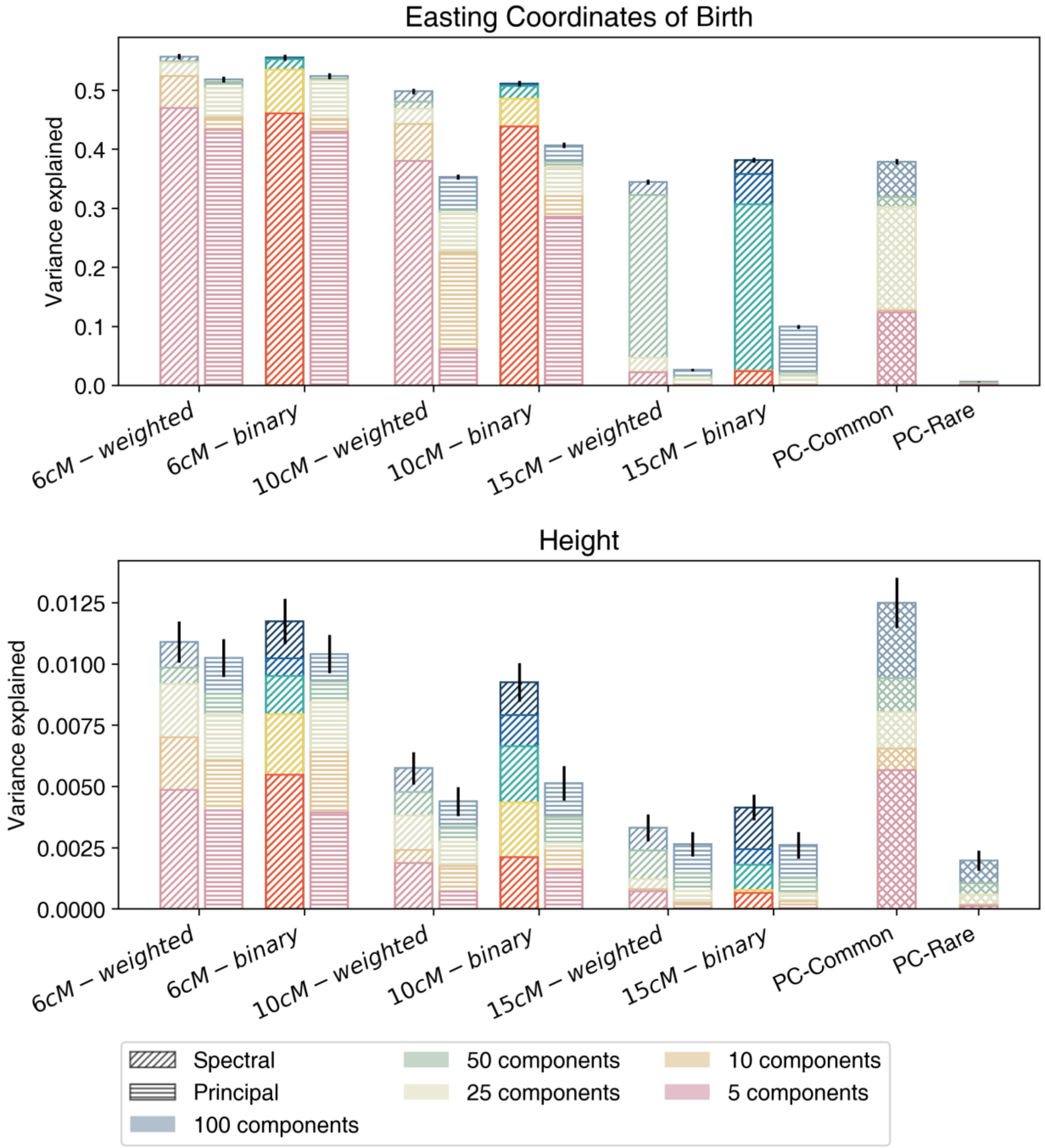

Supplemental Figure 2 – Proportion of variance in eastings and height explained by principal components in the UK Biobank. SPCs are highlighted in brighter colors. PCs of common and rare

variants are subscripted as PC-Common and PC-Rare, respectively. Compared to PCs, IBD-derived covariates explain a larger proportion of variance for both non heritable phenotypes; and have a similar performance on the heritable phenotype, especially for the first 25 principal components. Spectral components, including SPCs, perform equally or better than principal components of the IBD graph; especially if the graph is weighted.

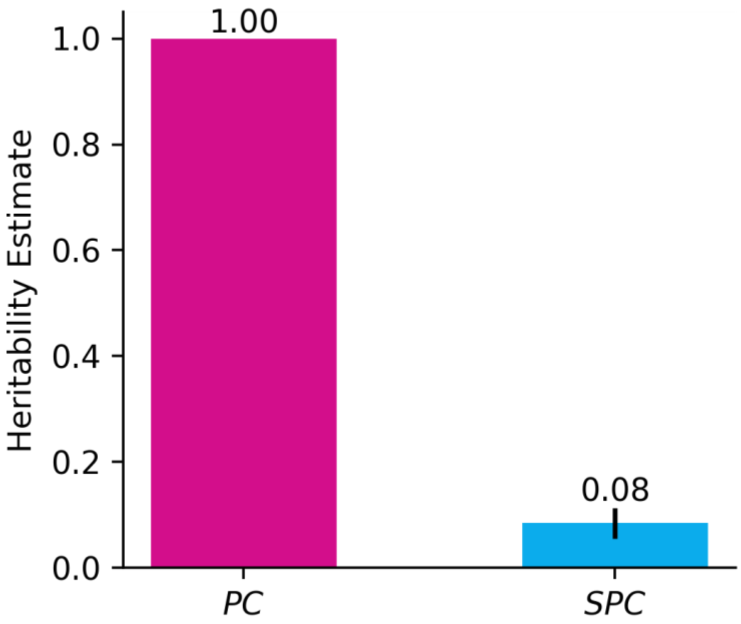

Supplemental Figure 3: narrow-sense heritability analysis aims to measure the proportion of heritability in a phenotype that is derived from genetic factors. Our analysis of a simulated ‘environmental smooth’ phenotype with no genetic effects using PCs or SPCs as covariates illustrates how SPCs are better suited for such analysis in phenotypes strongly affected by environmental factors. Adjusting for PCs results in a heritability estimate of 1.00 while adjusting for SPCs yields a much more realistic estimate of 0.08.

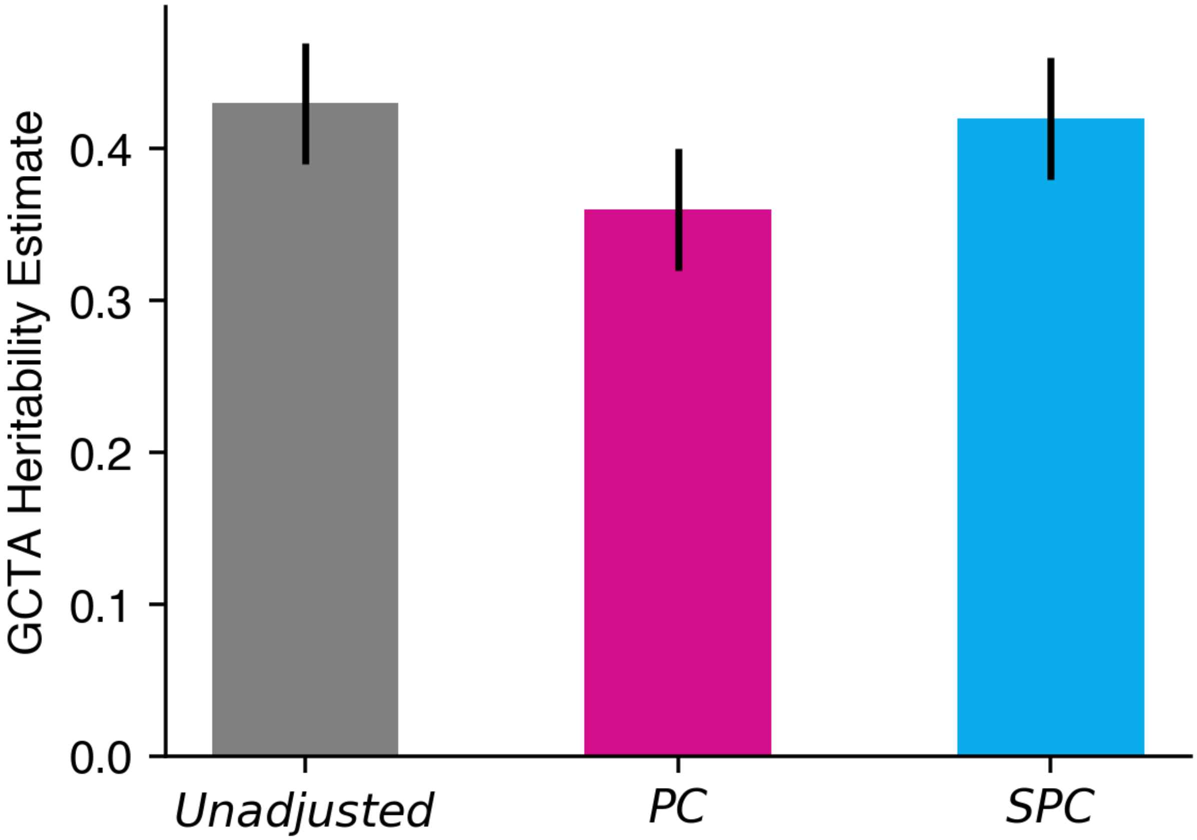

Supplemental Figure 4: narrow-sense heritability analysis aims to measure the proportion of heritability in a phenotype that is derived from genetic factors. Our analysis of a simulated phenotype with high heritability (h=0.8) and high polygenicity using PCs or SPCs as covariates illustrates how SPCs do not over-correct heritability estimates compared to PCs.

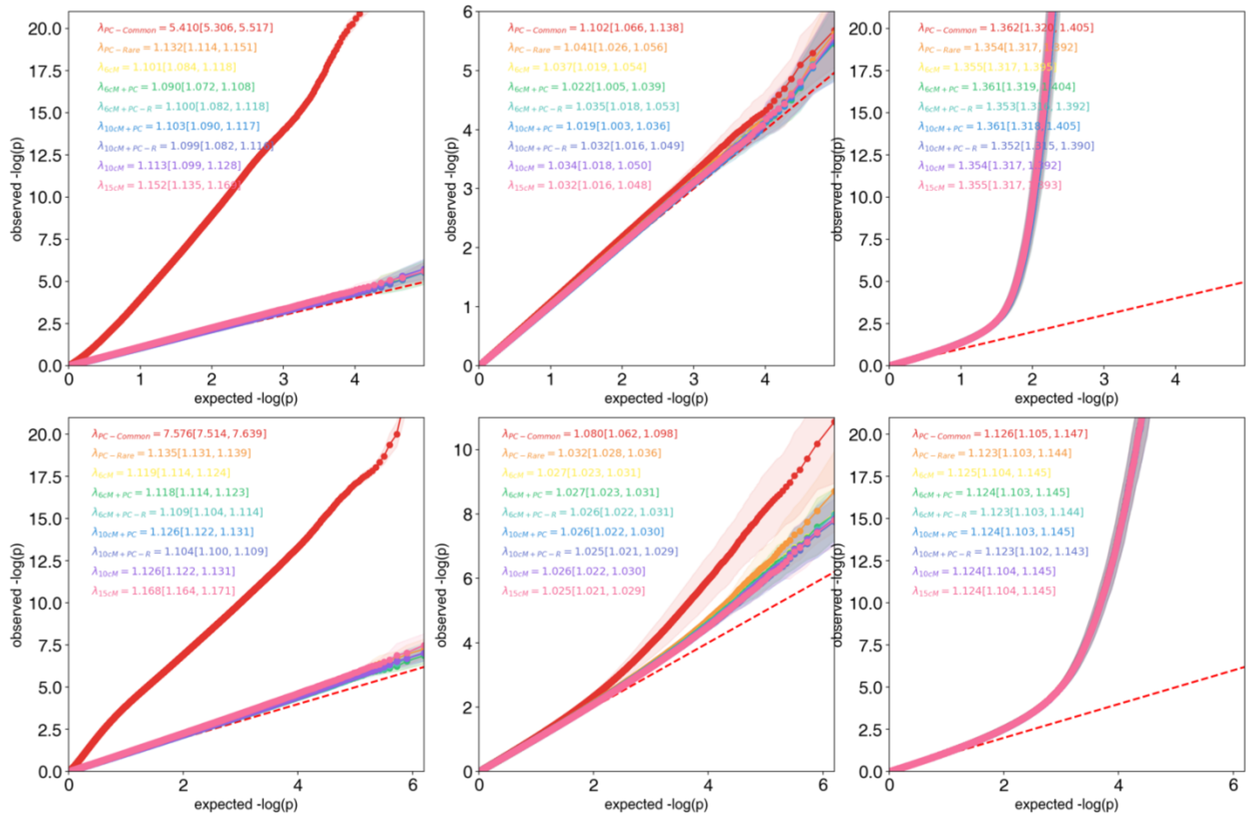

Supplemental Figure 5 - Genomic inflation of the results of GWAS analysis of simulated phenotypes in a simulated 5 by 5 grid of demes with 50,000 samples. 3 phenotypes were simulated. From left to right, environmental smooth, where the mean value of the phenotype amongst samples of the northernmost demes has a difference of 2σ with that of the southernmost demes, environmental sharp phenotype, where a single target deme has a phenotype with a non-zero mean distribution, while other demes have a zero mean distribution, and polygenic phenotypes, which are heritable with randomly chosen causal variants from the whole genome sequence data. The top panels show the inflation in the results of common variants GWAS, and the bottom panel show the results of uncommon variants (10<MAC<100) GWAS. SPCs outperform common and rare variant PCs in environmental smooth phenotype significantly, for both common, and rare-variant GWAS. SPCs outperform PCs of common variants in the common variant GWAS of the sharp phenotype, and both rare and common PC in the rare variant GWAS of the sharp phenotype. Their performance is similar to variant PCs in both GWAS analyses of the polygenic phenotype.

Supplemental methods

Performance against alternative approaches

We measured the efficacy of alternative strategies for adjusting for recent population structure by calculating the total proportion of variance explained in each phenotype by them.

Principal Components of rare variants

We calculated PCs of rare variants using two thresholds for rarity. First, all variants with minor allele frequency of less than 0.01. Second, variants with minor allele counts of 2-4 (Zaidi & Mathieson, 2020). We are only reporting the results of the first definition as it performed better in all scenarios in our simulations. We found the performance of rare variant PCs to be inconsistent compared to common variant PCs. Rare variant PCs explained a higher proportion of variance compared to PC in the environmental phenotypes (**Supplemental Figure 1**). Subsequently, the genomic inflation factor of in the GWAS of those phenotypes was lower when adjusted using rare PCs, both in the GWAS of common and rare variants (**Supplemental Figures 5 and 6**). However, they explained a lower proportion of variance in the polygenic phenotype, although that did not translate to any significant difference in the inflation of GWAS results. Rare variants lowered the heritability estimate of the environmental smooth phenotype, from 1.00 to 0.45, compared to PCs. However, they underperformed in comparison to SPCs across all of our simulated scenarios. Their performance in the analysis of the polygenic phenotype was not significantly different compared to SPCs. Using rare variant data available in the form WES data for 50,000 participants in the UK Biobanks, we found that PCs of common variants have a higher PVE for both eastings and height compared to PCs of rare variants (**Supplemental Figure 2**).

Alternative IBD-based covariates

We calculated 11 alternative IBD-based covariates. These alternative covariates varied from SPCs in three aspects. First, while SPCs are *spectral components* of the IBD relatedness, one can also calculate *principal components* of IBD relatedness. Second, the IBD relatedness can either be expresses as a binary or weighted relationship. Unlike the unweighted binary relationship, in the weighted IBD relatedness graphs, higher weights are assigned to edges connecting pairs of samples that share more than the minimum threshold of sharing. Finally, the minimum threshold of IBD sharing can itself be treated as a parameter. We heuristically chose three different minimum thresholds of 6cM, 10cM, and 15 cM. We used proportion of explained variance as the comparison criteria (**Supplemental Figures 1 and 2**).

Covariates generated using binary similarity matrix outperformed those derived from the weighted matrix when adjusting for the environmental phenotypes in simulation, and for both phenotypes in the UK Biobank, especially as the minimum IBD threshold was increased. Simultaneously, the latter group showed a higher correlation with PCs of common and rare variants, suggesting a higher level of overlap in the signals represented by the covariates.

Spectral components (including SPCs) outperformed principal components. The advantage of spectral components over principal components was small but maintained across both environmental scenarios. This difference was statistically significant for environmentally smooth phenotype, yet not significant for the sharp phenotype, most likely due to its nonlinear structure. The gap between SPCs and IBD-based principal components increases in the analysis of easting and height in UK Biobank, across all minimum length thresholds. The first 5 SPCs calculated

using a binary network with a minimum threshold of 10 cM had a higher PVE for eastings compared to the first 100 principal components calculated using the same network (**Supplemental Figure 2**). There was a significant difference between spectral components and principal components calculated from the weighted matrix, especially as the minimum IBD threshold is increased. SPCs have 67% higher performance at 6cM, 84% at 10cM, and 92% at 15cM when predictive the sharp phenotype.

Increasing the minimum threshold of IBD sharing used to generate the relatedness network had negligible effect on PVE in simulated phenotypes. Consequently, it did not significantly chance the inflation of p-values in the GWAS of the simulated phenotypes. However, in our analysis of PVE of height and eastings in UK Biobank, increasing this threshold significantly lowered PVE, to the point where the PVE of SPCs generated using the 15 cM network were lower than those of PCs in the analysis of eastings.

Graph structure captured by SPCs

Spectral components can recover non-linear properties in a graph. Here we will describe what that entails for the characteristics of the population structure they extract from IBD relatedness graphs. SPCs attribute a set of numerical values to each vertex based on its projections on the set of principal axes of variation in the graph. The level of detail represented by each axis depends on the corresponding eigenvalue association with it. Eigenvector associated with the smaller eigenvalues will assign similar number to neighbors on the graph, whereas the eigenvector associated with the larger eigenvalues will assign varying numbers to similar nodes. Thus, ignoring zero eigenvalues, the first axis, with the lowest eigenvalue attached to it will capture the most polarizing aspects of variations in the relatedness in the graph with lowest level of granularity. The first components before that, those with zero, or close to zero eigenvalues, incorporate a clustering of vertices into groups of recent genetic ancestry where participant from the major ancestry group $\boldsymbol{a}$ are represented by nonzero values with the mean $1/\sqrt{n_a}$, while other samples, with low, or no connections to this ancestry group, are represented by values closer to zero in that dimension. This dependency on connection, and not balance in representation is among the distinctions between SPCs and PCs (Lee et al., 2010). The number of highly distinctive features is thus derived from the number of distinct IBD families present in the dataset with heavy connections. An extreme example happens if the dataset is comprised of two heavily connected familial groups (founder populations) with little or no connections to each other. These groups are represented by their own dimension, even if they are not very well represented in the ascertainment. The SPCs also include dimensions that represent cross-family similarities. These dimensions have higher than zero eigenvalues associated with them. Thus, in the absence of strong clustering (i.e. a homogeneous cohort), The SPCs can still represent overlapping groups of individuals with recent genetic similarities.
